# Difference in Early Activation of NF-*κ*B and MCP-1 in Acinar-Cell-Rich versus Fibrotic Human Pancreas Exposed to Surgical Trauma and Hypoxia

**DOI:** 10.1155/2014/460363

**Published:** 2014-07-24

**Authors:** Matias Laaninen, Merja Bläuer, Juhani Sand, Isto Nordback, Johanna Laukkarinen

**Affiliations:** Department of Gastroenterology and Alimentary Tract Surgery, Tampere Pancreas Laboratory, Tampere University Hospital, Teiskontie 35, 33521 Tampere, Finland

## Abstract

*Objectives.* Previously we have shown that a pancreas with over 40% acinar cells is exposed to postoperative pancreatitis and other complications after pancreaticoduodenectomy (PD). Our aim was to analyze the expression of NF-*κ*B and MCP-1 in the cut edge of human pancreas after PD in both acinar-cell-rich and fibrotic pancreata. *Methods.* Several pancreatic samples from six patients, three with acinar-cell-rich and three with fibrotic pancreata, were exposed to surgical trauma in PD, and thereafter to hypoxemia for 15 minutes, 2–2.5 hours, 4 hours, or 6 hours, to mimic postoperative conditions of the pancreatic remnant in a patient. Immunohistochemical analysis of inflammation markers (NF-*κ*B, MCP-1) was performed. *Results.* In the acinar-cell-rich pancreata, intra-acinar NF-*κ*B and MCP-1 expression increased from mild at 15 minutes to high during the first 4 hours, whereas in ductal cells MCP-1 staining was highly intense at both time points. Acinar cell NF-*κ*B and MCP-1 expression and ductal cell MCP-1 expression were also observed in the fibrotic pancreata, but the activation remained low throughout the 6 hours. *Conclusions.* In acinar-cell-rich pancreas, an extensive inflammatory cascade begins almost immediately after surgical trauma. Fibrosis may limit the progression of inflammatory process in pancreas.

## 1. Introduction

Pancreaticoduodenectomy (PD) has become a standard operation with low mortality. However, at 42%–60% perioperative morbidity remains substantial. The most common complications include delayed gastric emptying, postoperative pancreatic fistula, wound infections, and postpancreatectomy hemorrhage [1–4].

A pancreas at high risk of severe complications can be predicted perioperatively. Acinar-cell-rich pancreas (defined as showing over 40% of acinar cells in the pancreatic transection line) is accompanied by an increased risk of postoperative pancreatitis or milder pancreatic irritation [3, 5, 6]. In our previous study [6], 92% of the patients with acinar-cell-rich pancreas developed postoperative complications. The complication rate decreased to 21% when there was more than 60% of fibrosis in the pancreatic transection line. We hypothesized that intraoperative pancreatic injury may immediately activate the inflammatory cascade in the remaining pancreas and that this activation may differ in acinar-cell-rich and fibrotic pancreas.

According to the prevailing theory, acute pancreatitis is set off by uncontrollable activation of trypsin leading to excitation of other digestive enzymes and, eventually, autodigestion and inflammation [7]. The inflammatory cascade, especially the signaling molecules involved, has been under intense scrutiny in recent years. Several signaling molecules have been shown to play important roles in the progression of the inflammation process in the pancreas. They include among others nuclear factor *κ*B (NF-*κ*B); monocyte chemoattractant protein 1 (MCP-1); interleukins IL-1, IL-2, and IL-6; platelet-activating factor (PAF); substance P; and tumor necrosis factor *α* (TNF-*α*) [8–10]. Both NF-*κ*B and MCP-1 are shown to upregulate early in acute pancreatitis [9–13]. In animal models, NF-*κ*B activates within 30 minutes and MCP-1 within 60 minutes in acinar cells after the induction of inflammation [11, 14] and this activation leads to exacerbation of acute pancreatitis [9, 15, 16].

The inflammatory cascade in human pancreas after surgical trauma has not been previously investigated. The aim of this study was to investigate postoperative inflammation in acinar-cell-rich and fibrotic human pancreas exposed to surgical trauma and hypoxia.

## 2. Materials and Methods

From among the patients undergoing PD in Tampere University Hospital, six individuals were chosen for the study based on the histopathology of the cut edge of the pancreas: three with acinar-cell-rich pancreas (>40% acini on the cut edge) and three with fibrotic pancreas (>60% fibrosis on the cut edge). In the acinar-cell-rich group, the patients and the final histopathological diagnoses were as follows: 50-year-old female with neuroendocrine carcinoma of the head of the pancreas, 55- and 57-year-old males with adenocarcinomas of the head of the pancreas. In the fibrotic group, the diagnoses were as follows: 78-year-old male with serous cystadenoma of the head of the pancreas, 60- and 74-year-old males with adenocarcinomas of the head of the pancreas.

During the operation, at the time of the transection, a tissue sample (size 2 mm thick, 10 mm in diameter) was harvested from the cut edge. The specimen was cut into five pieces which were immersed in physiologic NaCl solution to prevent them from drying. The tissue was thus exposed to surgical trauma followed by ischemia ex vivo, in an endeavor to mimic the conditions at the cut edge of the pancreatic remnant in the patient. At 15 minutes, 2–2.5 hours, 4 hours, or 6 hours, the NaCl solution was replaced by 4% paraformaldehyde and the samples were allowed to fix overnight. The samples were then dehydrated and embedded in paraffin. Sections (5 *μ*m thick) were cut for immunohistochemical analysis.

Immunohistochemical analysis was performed using the following antibodies at the dilutions indicated: anti-NF-*κ*B p50 (1 : 200; AbD Serotec, Oxford, UK) and anti-MCP-1 (1 : 200; AbD Serotec). Controls included omission of the primary antibodies and the use of nonimmunized mouse and rabbit IgG. The staining was performed with a broad-spectrum Histostain-Plus kit (Invitrogen, Camarillo, CA, USA) as previously described [17]. The sections were lightly counterstained with hematoxylin.

The slides were then subjected to microscopic analysis (Nikon Microphot-FXA). Quantitative analysis of NF-*κ*B 15-minute and 4-hour samples was performed by two independent researchers (ML, MB). The percentage of activated acinar cells (stained nucleus) out of the total number of acini in each sample was determined from representative areas using a magnification of 250. The means (±SEM) of the three acinar-cell-rich and the three fibrotic samples were then calculated. Differences in the intensity of MCP-1 staining were determined semiquantitatively and expressed as low, moderate, or high.

The study protocol was approved by the ethics committee of Tampere University Hospital.

## 3. Results

**Figure 1:**
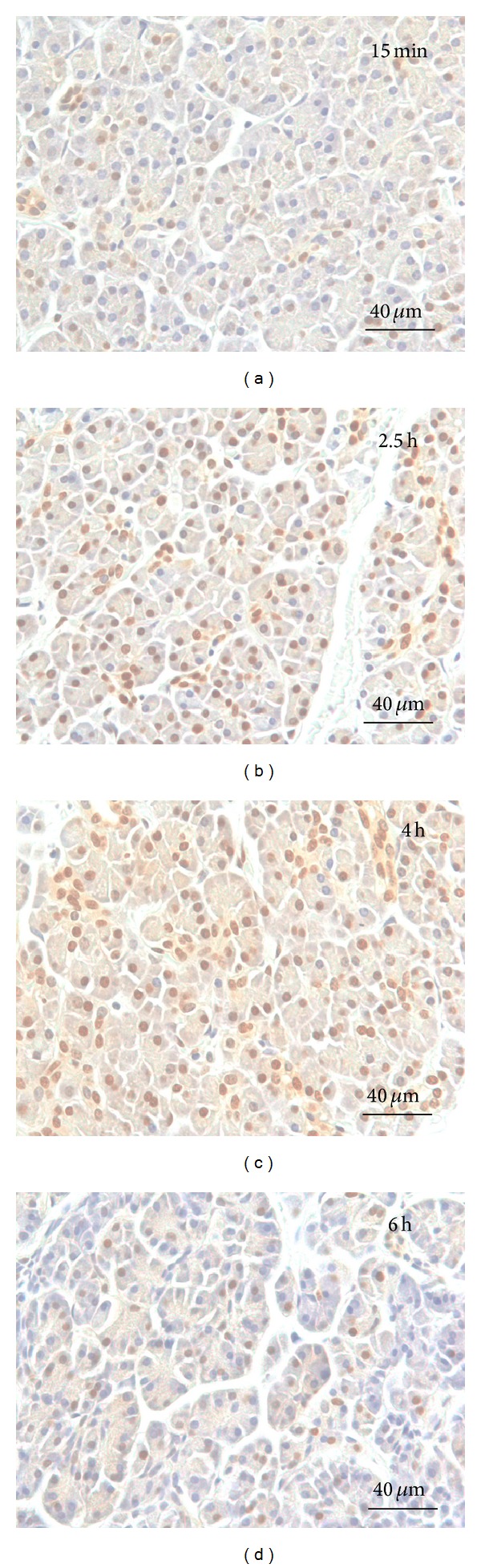
NF-*κ*B activation in acinar-cell-rich pancreas. Immediately after sampling, parallel portions of each tissue specimen were immersed in saline and kept at room temperature for 15 minutes (a), 2.5 hours (b), 4 hours (c), or 6 hours (d), after which they were fixed and processed for immunohistochemical analysis. The slides were counterstained with hematoxylin. Slight staining of acinar cell nuclei can be seen at 15 minutes (a) and significant amplification is observed at 2.5 hours (b). Almost every acinar cell nucleus is stained at 4 hours (c) and activation decreases at 6 hours (d).

**Figure 2:**
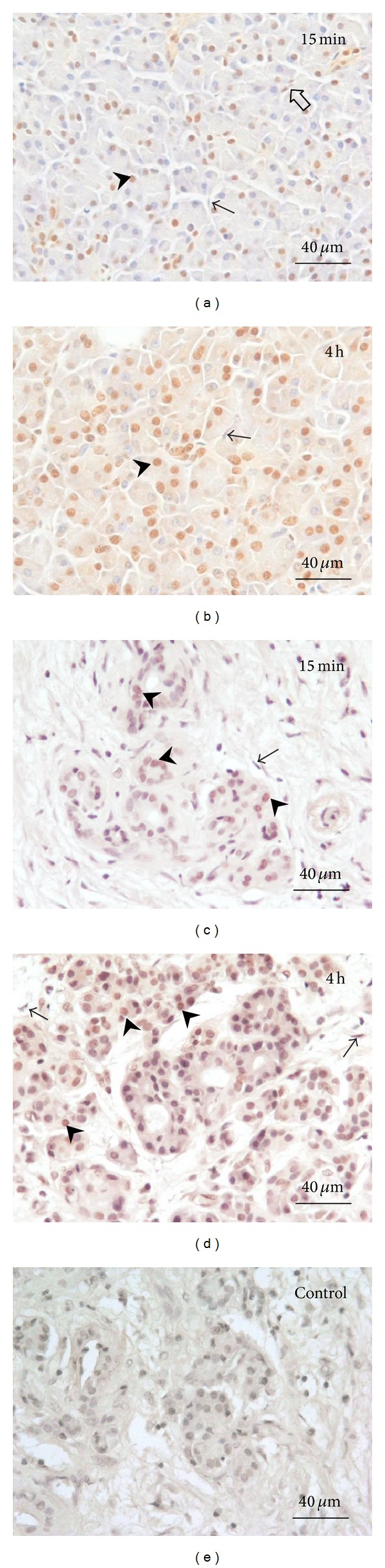
NF-*κ*B expression in acinar-cell-rich ((a), (b)) and fibrotic ((c), (d)) pancreata. (a) and (c) are 15-minute sample and (b) and (d) represent 4-hour time points. Arrowheads indicate representative NF-*κ*B-expressing nuclei in acinar cells. NF-*κ*B-positive fibroblasts were rare (open arrow in (a)), the fibroblast nuclei being predominantly negative (arrows). The increase in NF-*κ*B activation is more prominent in acinar-cell-rich pancreata ((a) and (b)) than in fibrotic pancreata ((c) and (d)). Control stainings were negative (e).

NF-*κ*B staining was seen in the nuclei of acinar cells, and MCP-1 activation was found in the cytoplasms of acini and ductal cells. Qualitative analysis revealed the progression of NF-*κ*B activation in acinar-cell-rich pancreata during the 6-hour period (Figure 1) such that the highest NF-*κ*B expression was at 4 hours (Figures 1 and 2). In the fibrotic pancreata, acinar cell activation of NF-*κ*B was also detected, but the tissue expression of NF-*κ*B did not increase over time (Figure 2). NF-*κ*B-positive fibroblasts were scarce, with the fibroblast nuclei being predominantly unstained. In all tissue sections the intensity of NF-*κ*B staining appeared even and no gradient from outside to inside was detectable.

**Figure 3:**
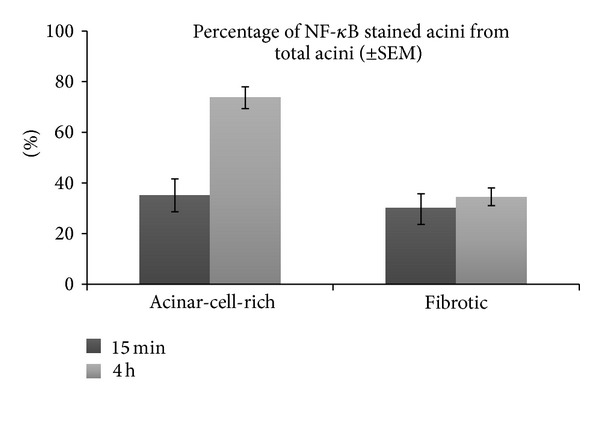
Comparison of NF-*κ*B activation in acinar-cell-rich and fibrotic pancreata. The means (±SEM) of the three acinar-cell-rich and the three fibrotic samples were calculated and then compared at 15 minutes and 4 hours. In acinar-cell-rich pancreata, a significant increase in NF-*κ*B expression occurs between 15 minutes (35%) and 4 hours (74%). In fibrotic pancreata, the change between 15 minutes (30%) and 4 hours (35%) is minor.

Quantitative analysis for the acinar-cell-rich pancreata showed that acinar cell NF-*κ*B activation increased from mild at 15 minutes (35% ± 7%, mean ± SEM) to high (74% ± 4%) during the first 4 hours (Figure 3). NF-*κ*B activation was 30% (±6%) at 15 minutes and 35% (±4%) at 4 hours in the fibrotic pancreata (Figure 3).

**Figure 4:**
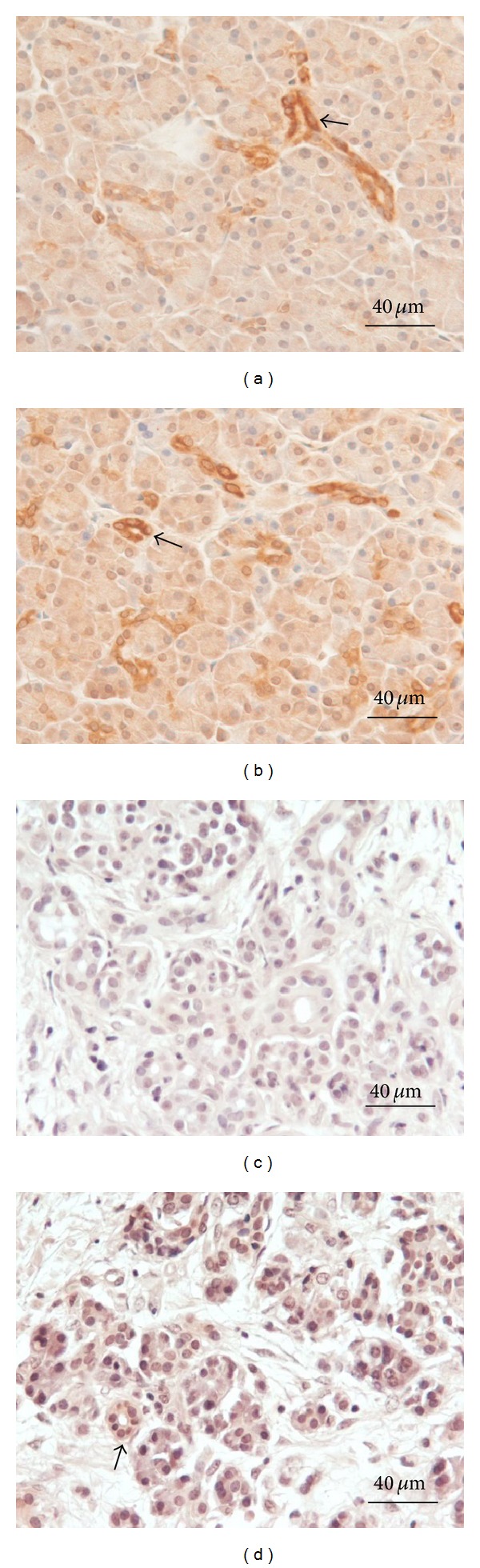
MCP-1 expression in acinar-cell-rich ((a), (b)) and fibrotic ((c), (d)) pancreata. Immediately after sampling, parallel portions of each tissue specimen were immersed in saline and kept at room temperature for 15 minutes ((a), (c)) or 4 hours ((b), (d)), after which they were fixed and processed for immunohistochemical analysis. MCP-1 staining was equally intense in the ductal cells of acinar-cell-rich pancreata after 15 minutes and 4 hours ((a), (b), arrows), whereas intra-acinar MCP-1 expression was observed to slightly increase with time. In fibrotic pancreata, MCP-1 in ductal and acinar cells remained undetectable at 15 minutes (c). At 4 hours, weak staining can be detected in ductal cells ((d), arrow). The slides were counterstained with hematoxylin.

Acinar cell expression of MCP-1 increased from low at 15 minutes to moderate during the first 4 hours in the acinar-cell-rich pancreata, whereas in ductal cells MCP-1 staining was highly intense at both time points (Figure 4). Acini and ductal cells did not express MCP-1 at 15 minutes in the fibrotic pancreata and only minor staining was observed at 4 hours (Figure 4).

## 4. Discussion

An acinar-cell-rich pancreas is at higher risk of post-PD complications than is a fibrotic pancreas. Intraoperative pancreatic injury may activate the inflammatory cascade differently in acinar-cell-rich pancreas and fibrotic pancreas. The role of inflammation markers in human pancreas following surgical trauma has not been previously studied and was the focus of this study. It was concluded that the intra-acinar cell inflammatory cascade may lead to pancreatitis almost immediately after induction of injury by surgical trauma and ischemia in acinar-cell-rich human pancreas, whereas fibrosis may limit the progression of inflammation in pancreas.

Several signaling molecules (such as IL-1, IL-2, IL-6, PAF, substance P, TNF-*α*, MCP-1, and NF-*κ*B) have been shown to play important roles in the progression of experimental acute pancreatitis [8–10]. Studies have shown that both NF-*κ*B and MCP-1 upregulate early in acute pancreatitis and may exacerbate its severity [9–16, 18, 19], which is why these markers were chosen for our study.

NF-*κ*B has been shown to regulate the transcription of several genes involved in immunity and inflammation [9]. Numerous studies have demonstrated an early and significant activation of pancreatic NF-*κ*B when acute experimental pancreatitis is induced in rats or mice using agents such as cerulein, taurocholate, and bile-pancreatic duct ligation [9, 11–13]. Acinar cells are considered to play a key role especially in early (within 30 minutes) pancreatic NF-*κ*B activation in experimental acute pancreatitis [11]. Activation of NF-*κ*B is followed by an increased number of proinflammatory cytokines and influx of inflammatory cells into the pancreas, leading to exacerbation of pancreatitis [9]. The importance of NF-*κ*B in the inflammatory process is substantiated by the fact that inhibiting its activation using antioxidants (e.g.,* N*-acetylcysteine) or anti-inflammatory agents (e.g., peroxisome proliferator-activated receptor *γ*, PPAR*γ*) has been shown to reduce the severity of pancreatitis in animal models [9, 15, 16, 18].

MCP-1 has been associated with several inflammatory diseases, including pancreatitis. Monocytes, T-lymphocytes, acinar cells, and stellate cells have all been shown to express MCP-1, and MCP-1 has been seen to upregulate in acute and chronic pancreatitis [10]. Acini express MCP-1 as early as 60 minutes after induction of acute experimental pancreatitis [14]. The importance of MCP-1 in the pathogenesis of pancreatic inflammation was substantiated in a study by Zhao et al. [19], where pancreatic inflammation and fibrosis was significantly reduced in rats with experimental chronic pancreatitis by giving them antichemokine gene therapy. In a study by Ishibashi et al. [16] the severity of acute pancreatitis was attenuated by blocking MCP-1 activity in rat models.

Knowledge about the role of acinar cells in the pathogenesis of acute pancreatitis has progressed over recent years. It has been suggested that acinar cells can act in the same manner as inflammatory cells. The latest studies show that the acini may be promoters of the inflammatory cascade. They secrete cytokines, chemokines, and adhesion molecules, resulting in activation and recruitment of circulating leukocytes [20, 21].

The consistency of the pancreas has been shown to affect the risk of post-PD complications. A soft pancreas and a small pancreatic duct diameter are known to increase morbidity [22, 23]. Postoperative pancreatitis, or subclinical pancreatic irritation, has recently been noted as a precursor of postoperative complications such as delayed gastric emptying and postoperative pancreatic fistula [3, 6]. In animal models, any injury to pancreatic parenchyma with scalpel or sutures has been shown to initiate an inflammatory process in the parenchyma that spreads throughout the pancreas [24, 25]. In our previous study, patients with acinar-cell-rich pancreas developed massive postoperative inflammation that exposed them to clinically significant complications [6]. So as far as we know, molecular-level events related to post-PD pancreatitis in the remnant of pancreas after PD have not been studied before.

In the postoperative state the pancreatic remnant suffers from hypoxia to some extent but not from* total* ischemia as in our ex vivo study. We recognize that this study therefore does not perfectly mimic postoperative conditions in the patient. Hypoxia has been shown to be an independent inducer of acute pancreatitis [26] and presumably acts as an aggravating factor for surgically induced pancreatic inflammation. The intensity of acinar cell activation may therefore be magnified in this setting. Hypoxia-induced acinar cell necrosis may also explain the decreased activation of NF-*κ*B at 6 h samples (Figure 1(d)), which is why we decided to use 4 h samples in our quantitative analyses.

In this study we found that in the acinar-cell-rich pancreata, acinar cell NF-*κ*B and MCP-1 activation increased from mild at 15 minutes to high after the first 4 hours, and ductal MCP-1 expression was highly intense at both time points. In the fibrotic pancreata, acinar cell expression of NF-*κ*B and MCP-1 and also ductal cell expression of MCP-1 were detected at the 6-hour monitoring, but the tissue expression of these markers remained lower. Our findings of the limiting role of fibrosis in pancreatic inflammation are also in line with a recent study of Acharya and colleagues [27], where fibrosis was seen to reduce acinar cell necrosis among patients with acute-on-chronic pancreatitis.

Whether and how fast the inflammation exacerbates into clinically relevant pancreatic inflammation or even pancreatitis are not known. However, in our previous study we showed that it is patients with acinar-cell-rich pancreas who develop clinically relevant pancreatic inflammation [6].

## 5. Conclusions

We hypothesize that a patient undergoing PD who has a large amount of acinar cells in the transection line (i.e., in the pancreatic remnant) is at high risk of developing a massive postoperative inflammatory cascade in the pancreas. The first 4 hours after the induction of surgical trauma may play an important role in the patient's postoperative prognosis. As postoperative pancreatitis often precedes other complications after PD, future therapeutic strategies targeting postoperative complications could consider anti-inflammatory treatments and could also focus them on perioperative—not just postoperative—treatment.

## References

[1] Büchler MW, Friess H, Wagner M, Kulli C, Wagener V, Z'Graggen K (2000). Pancreatic fistula after pancreatic head resection. *British Journal of Surgery*.

[2] Diener MK, Knaebel HP, Heukaufer C, Antes G, Büchler MW, Seiler CM (2007). A systematic review and meta-analysis of pylorus-preserving versus classical pancreaticoduodenectomy for surgical treatment of periampullary and pancreatic carcinoma. *Annals of Surgery*.

[3] Räty S, Sand J, Lantto E, Nordback I (2006). Postoperative acute pancreatitis as a major determinant of postoperative delayed gastric emptying after pancreaticoduodenectomy. *Journal of Gastrointestinal Surgery*.

[4] Ho V, Heslin MJ (2003). Effects of hospital volume and experience on in-hospital mortality for pancreaticoduodenectomy. *Annals of Surgery*.

[5] Räty S, Sand J, Nordback I (2007). Detection of postoperative pancreatitis after pancreatic surgery by urine trypsinogen strip test. *British Journal of Surgery*.

[6] Laaninen M, Bläuer M, Vasama K (2012). The risk for immediate postoperative complications after pancreaticoduodenectomy is increased by high frequency of acinar cells and decreased by prevalent fibrosis of the cut edge of pancreas. *Pancreas*.

[7] Frossard JL, Steer ML, Pastor CM (2008). Acute pancreatitis. *The Lancet*.

[8] Pandol SJ, Saluja AK, Imrie CW, Banks PA (2007). Acute pancreatitis: bench to the bedside. *Gastroenterology*.

[9] Rakonczay Z, Hegyi P, Takács T, McCarroll J, Saluja AK (2008). The role of NF-*κ*B activation in the pathogenesis of acute pancreatitis. *Gut*.

[10] Marra F (2005). Renaming cytokines: MCP-1, major chemokine in pancreatitis. *Gut*.

[11] Gukovsky I, Gukovskaya AS, Blinman TA, Zaninovic V, Pandol SJ (1998). Early NF-*κ*B activation is associated with hormone-induced pancreatitis. *American Journal of Physiology—Gastrointestinal and Liver Physiology*.

[12] Telek G, Ducroc R, Scoazec JY, Pasquier C, Feldmann G, Rozé C (2001). Differential upregulation of cellular adhesion molecules at the sites of oxidative stress in experimental acute pancreatitis. *Journal of Surgical Research*.

[13] Samuel I, Yorek MA, Zaheer A, Fisher RA (2006). Bile-pancreatic juice exclusion promotes Akt /NF- kappaB activation and chemokine production in ligation-induced acute pancreatitis. *Journal of Gastrointestinal Surgery*.

[14] Grady T, Liang P, Ernst SA, Logsdon CD (1997). Chemokine gene expression in rat pancreatic acinar cells is an early event associated with acute pancreatitis. *Gastroenterology*.

[15] Vaquero E, Gukovsky I, Zaninovic V, Gukovskaya AS, Pandol SJ (2001). Localized pancreatic NF-*κ*B activation and inflammatory response in taurocholate-induced pancreatitis. *American Journal of Physiology: Gastrointestinal and Liver Physiology*.

[16] Ishibashi T, Zhao H, Kawabe K (2008). Blocking of monocyte chemoattractant protein-1 (MCP-1) activity attenuates the severity of acute pancreatitis in rats. *Journal of Gastroenterology*.

[17] Bläuer M, Heinonen PK, Martikainen PM, Tomás E, Ylikomi T (2005). A novel organotypic culture model for normal human endometrium: regulation of epithelial cell proliferation by estradiol and medroxyprogesterone acetate. *Human Reproduction*.

[18] Hashimoto K, Ethridge RT, Saito H, Rajaraman S, Evers BM (2003). The PPAR*γ* ligand, 15d-PGJ2, attenuates the severity of cerulein-induced acute pancreatitis. *Pancreas*.

[19] Zhao HF, Ito T, Gibo J (2005). Anti-monocyte chemoattractant protein 1 gene therapy attenuates experimental chronic pancreatitis induced by dibutyltin dichloride in rats. *Gut*.

[20] Dios ID (2010). Inflammatory role of the acinar cells during acute pancreatitis. *World Journal of Gastrointestinal Pharmacology and Therapeutics*.

[21] Vonlaufen A, Apte MV, Imhof BA, Frossard JL (2007). The role of inflammatory and parenchymal cells in acute pancreatitis. *Journal of Pathology*.

[22] Pratt WB, Callery MP, Vollmer CM (2008). Risk prediction for development of pancreatic fistula using the ISGPF classification scheme. *World Journal of Surgery*.

[23] Ansorge C, Strömmer L, Andrén-Sandberg Å, Lundell L, Herrington MK, Segersvärd R (2012). Structured intraoperative assessment of pancreatic gland characteristics in predicting complications after pancreaticoduodenectomy. *British Journal of Surgery*.

[24] Lämsä T, Jin HT, Nordback PH (2009). Pancreatic injury response is different depending on the method of resecting the parenchyma. *Journal of Surgical Research*.

[25] Lämsä T, Jin H, Nordback PH, Sand J, Nordback I (2008). Effects of diameter, number and tightness of sutures on pancreatic injury response. *Digestive Surgery*.

[26] Hackert T, Hartwig W, Fritz S, Schneider L, Strobel O, Werner J (2009). Ischemic acute pancreatitis: clinical features of 11 patients and review of the literature. *The American Journal of Surgery*.

[27] Acharya C, Cline RA, Jaligama D (2013). Fibrosis reduces severity of acute-on-chronic pancreatitis in humans. *Gastroenterology*.

